# Cellular response to persistent foot-and-mouth disease virus infection is linked to specific types of alterations in the host cell transcriptome

**DOI:** 10.1038/s41598-018-23478-0

**Published:** 2018-03-22

**Authors:** Lingling Han, Xiu Xin, Hailong Wang, Jiadai Li, Yao Hao, Mingzhen Wang, Congyi Zheng, Chao Shen

**Affiliations:** 1 State Key Laboratory of Virology, College of Life Sciences, Wuhan University, Wuhan, China; 2 China Center for Type Culture Collection, Wuhan University, Wuhan, China

## Abstract

Food-and-mouth disease virus (FMDV) is a highly contagious virus that seriously threatens the development of animal husbandry. Although persistent FMDV infection can dramatically worsen the situation, the mechanisms involved in persistent FMDV infection remain unclear. In the present study, we identified the presence of evolved cells in the persistently FMDV-infected cell line. These cells exhibited resistance to the parent FMDV and re-established persistent infection when infected with FMDV-Op (virus supernatant of persistent infection cell lines), emphasizing the decisive role of evolved host cells in the establishment of persistent FMDV infection. Using RNA-seq, we identified the gene expression profiles of these evolved host cells. In total, 4,686 genes were differentially expressed in evolved cells compared with normal cells, with these genes being involved in metabolic processes, cell cycle, and cellular protein catabolic processes. In addition, 1,229 alternative splicing events, especially skipped exon events, were induced in evolved cells. Moreover, evolved cells exhibited a stronger immune defensive response and weaker MAPK signal response than normal cells. This comprehensive transcriptome analysis of evolved host cells lays the foundation for further investigations of the molecular mechanisms of persistent FMDV infection and screening for genes resistant to FMDV infection.

## Introduction

Foot-and-mouth disease virus (FMDV) is an 8.5-kb single-stranded positive-sense RNA virus of the family Picornaviridae and genus Aphthovirus that affects all cloven-footed animals. Infection presents as vesicle formation on the mouth and hooves followed by skin erosions of the cutaneous mucosa; it is accompanied by symptoms of fever, weight loss, lameness, and salivation^1^. FMDV in livestock often results in substantial economic losses and social impacts, including loss of production, costly control measures, and limits on international trade of livestock and related products^2,3^. However, apart from causing acute infection and disease, under certain circumstances the virus can adopt an asymptomatic carrier state, even in vaccinated ruminants exposed to the live virus^4–6^. Such carriers can cause re-outbreak of foot-and-mouth disease, making control efforts even more troublesome and costly^5,7^.

Currently, the mechanisms by which FMDV persistence is established and maintained are not fully understood, although it has been suggested that both cellular and humoral immune responses as well as cytokine responses play critical roles. For other virus-cell systems, aspects of the host cell including mutations, reduced expression of viral receptors^8–11^, obstacles to viral uptake after receptor events^12^, and changes in immune response including cellular immunity, humoral immunity, and cytokine response^13^ contribute to the establishment of a carrier state infection *in vitro*. However, genetic changes of the virus including production of defective interfering particles^14^, acquisition of new epitopes^15^, the occurrence of replication-defective mutations^16^, and the emergence of variants that replicate with new replication intermediates^17^ have also been reported. However, these mutations do not seem to contribute to the establishment of persistent FMDV infection.

As an RNA virus, FMDV utilizes the host cell to complete its replication cycle; most steps in virus infection involve interactions between viral components and host factors. On one hand, FMDV utilizes host cellular machinery to complete virus genome replication and associated infection events; numerous host factors are hijacked to complete steps such as assembling the viral RNA replication complex, selecting and recruiting viral RNA replication templates, and activating the complex for RNA synthesis^18–20^. On the other hand, the host cell activates a defense mechanism to limit viral growth to promote cell survival^21,22^. Interactions between the virus and its host determine the ultimate outcome of the battle for survival, which depends on whether the virus is inhibited and gradually cleared by the host cells, enters a lytic infection, or becomes suppressed and enters persistent infection. In 1985, de la Torre *et al*. established an *in vitro* model of persistently infected cell lines with an FMDV of serotype C (clone C-S8c1)^23^. Using this model, they showed that co-evolution of host cells and viruses occur during persistent FMDV infection^24^. Their subsequent studies suggest that the evolution of host cells, rather than viruses, plays a decisive role; that is, the critical element in the establishment of persistent FMDV infection of BHK-21 cells is the ability of host cells to vary genetically and phenotypically, which promotes the selection of cells with increased resistance to the virus^25,26^. Coincidentally, an *in vitro* model based on FMDV O-type persistence of bovine-derived primary cells also exhibits virus-host co-adaptation^27^. Together, these results indicate that during persistent FMDV infection, the virus interacts with host cells and undergoes co-evolution, during which changes in host cells play a decisive role in the establishment of persistent infection.

However, the molecular mechanisms involved in host-directed persistence of FMDV and antiviral responses remain poorly understood. There is limited information regarding specific changes that occur in host cells and the significance of these changes for persistent FMDV infection. Many of these changes can be reflected by alterations in the transcriptome of host cells. Early studies in persistently FMDV-infected cattle using bovine transcriptome microarray led to the discovery of several genes and pathways that are differentially expressed in the carrier^28,29^. However, all of these studies were conducted using limited genome coverage DNA microarrays, which may miss many important genes. In addition, these studies cannot analyze other types of changes, such as alternative splicing (AS). Here, we isolated evolved host cells (BHK-VECs) from persistent FMDV serotype O-infected BHK-21 cells (named BHK-Op cells). We found that BHK-VECs resisted infection of FMDV and FMDV-Op. Moreover, the infection of these evolved host cells with FMDV-Op resulted in re-establishment of persistent infection. We also found that many genes involved in cell metabolism, cell cycle, and protein metabolism were differentially expressed between BHK-VECs and BHK-21 cells, and 1,229 AS events, particularly skipped exon events, were induced in BHK-VECs. Moreover, BHK-VECs showed a stronger immune defensive response and weaker MAPK signal response than BHK-21 cells.

To date, there are no relevant reports regarding how FMDV affects gene expression in host cells and host cell RNA splicing at the transcriptome level during persistent infection. Our study not only serves as a basis for further studies on the transcriptome of persistent FMDV-infected host cells but also facilitates the discovery of candidate genes resistant to FMDV infection.

## Results and Discussion

### Emergence of FMDV-negative cells which were resistant to the infection of FMDV during persistent infection

An important role of host cells in the initiation and maintenance of FMDV persistence has previously been demonstrated in cell culture^25^. Early studies of persistently FMDV C-S8c1-infected Cl-BHK-Rcl cells show that host cells exhibit extensive heterogeneity during persistent infection and become increasingly resistant to FMDV^24,26^. However, virus antigens show cell density-dependent expression during persistent FMDV infection^30^. This raises the possibility that antigen-negative cells among persistently infected cells are the result of cell evolution resulting in increased resistance to FMDV infection.

**Figure 1:**
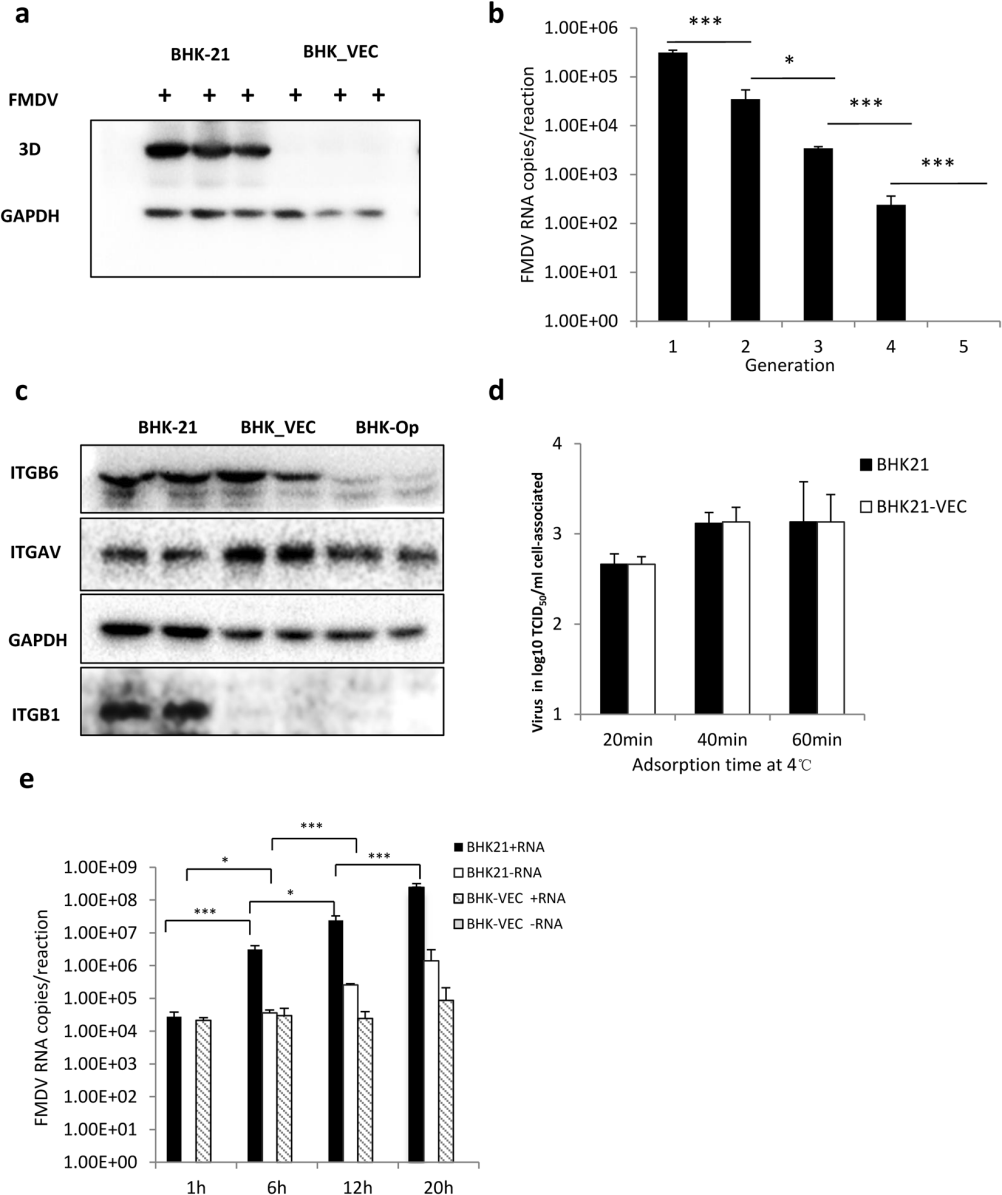
Emergence of FMDV-resistant cells during persistent infection. (**a**) BHK-21 cells and BHK-VECs were infected with FMDV at 2.5 × 10^−4^ PFU/cell for 18 h, and whole-cell extracts were analyzed by western blotting using rabbit polyclonal anti-3D antibody and mouse monoclonal anti-GAPDH. (**b**) BHK-VECs were infected with FMDV at 5 × 10^−2^ PFU/cell. Infected cells were subcultured, and intracellular viral RNA of each generation was measured using qRT-PCR. (**c**) Expression of FMDV integrin receptors (ITGB6, ITGAV, ITGB1) in BHK-VEC, BHK-Op, and uninfected BHK-21 cell cultures were detected by western blotting using specific antibodies. (**d**) Adsorption of FMDV to BHK-21 cells and BHK-VECs. BHK21 cells and BHK-VECs were infected with FMDV at 2.8 PFU/cell and maintained at 0–4 °C. At the indicated time point, supernatant was removed rapidly, and monolayers were washed with cold PBS three times. One mL serum-free MEM was added to the monolayers. Cell suspensions were then subjected to three cycles of freezing and thawing and centrifuged for 5 min at 12,000 × g at 4 °C. Supernatant was used to quantify the bound virus titer by TCID_50_ assay. (**e**) Detection of intracellular viral RNA replication in BHK-VECs and BHK-21 cells. BHK21 cells and BHK-VECs were infected with FMDV at 2.5 × 10^−4^ PFU/cell. At the indicated time points, intracellular RNA was isolated, and intracellular virus RNA (positive-stranded and negative-stranded RNA) was quantified by qRT-PCR. ***p < 0.001, *p < 0.05.

To confirm this possibility, we used the cell line BHK-Op, which is persistently infected with FMDV O-Akesu/58, to study persistent FMDV infection^31^. We first confirmed the cell density-dependent expression of virus antigens in persistently FMDV-infected cells by indirect immunofluorescence assay. We observed that as few as 2% of persistently infected BHK-Op43 cells (persistently infected BHK-21 cells after 43 passages) were FMDV 3D-positive, whereas most BHK-Op43 cells were FMDV 3D-negative (Fig. S1), consistent with previous reports^30^. Isolation of these 3D-negative cells by gradient dilution and quantitative real-time polymerase chain reaction (qRT-PCR) showed that they were free of FMDV RNA (Supplementary Table S1); these cells were named BHK-VECs. To determine whether these FMDV-negative cells could be infected by FMDV, they were inoculated, along with parental BHK-21 cells, with FMDV for 18 h at 2.5 × 10^−4^ plaque-forming units (PFU)/cell. Cells were harvested, and cell lysates were analyzed by western blotting for FMDV 3D protein. We found that infected BHK-21 cells exhibited significant cytopathic effect, whereas BHK-VECs incubated with FMDV showed no sign of cytopathic effect. Also, in contrast to infected BHK-21 cells, no 3D protein was detected in infected BHK-VECs (Fig. 1a). To further examine these details, we next inoculated BHK-VECs with FMDV at 5 × 10^−2^ PFU/cell. When infected cells reached 95% confluence, half of the cells were used for passaging, and the other half were collected and lysed to quantify virus RNA using qRT-PCR; this was performed for five generations. We found that FMDV gradually decreased in infected BHK-VECs with their passage until no FMDV RNA was detectable at the 5^th^ generation (Fig. 1b). These results suggest that although native BHK-21 cells were efficiently infected by FMDV, FMDV-negative cells among the persistently infected cells appeared resistant to FMDV infection.

As BHK-VECs exhibited a non-permissibility of FMDV infection, we next tested whether expression of the viral receptor was impaired. The same number of BHK-21, BHK-VEC, and BHK-Op cells were collected, and cell lysates were analyzed using western blotting for ITGB6, ITGAV, ITGB1, and ITGB3 protein. We found no differences in ITGAV expression among the three cell lines or ITGB6 expression between BHK-21 cells and BHK-VECs (Fig. 1c). However, ITGB6 expression was markedly reduced in BHK-Op cells, suggesting that replication of the virus down-regulated ITGB6 expression in host cells during persistent FMDV infection. Similarly, the expression of ITGB1 was markedly reduced in BHK-VECs and BHK-Op cells compared with BHK-21 cells. ITGB3 was not detected. To verify whether these differentially expressed receptors accounted for the resistance of BHK-VECs to FMDV infection, we measured cell attachment of the virus. The kinetics of FMDV attachment, as measured by the association of PFU to cell monolayers, were not significantly different between BHK-21 cells and BHK-VECs (Fig. 1d), implying that the reduction in virus yields of BHK-VECs occurred after virus attachment.

Next, we determined whether the resistance of FMDV-negative cells among persistently infected cells was due to a blockade of intracellular viral replication. Cultures of the same number of BHK-21 cells and BHK-VECs were infected with FMDV, and intracellular RNA was isolated at different time points post-infection to quantify intracellular virus RNA (positive- and negative-stranded RNA) using qRT-PCR. We found that more virus positive-stranded and negative-stranded RNA was detected in FMDV-infected BHK-21 cells than in FMDV-infected BHK-VECs (Fig. 1e). Intracellular FMDV positive-stranded RNA levels in BHK-VECs failed to increase significantly over time, and no FMDV-negative-stranded RNA was detected throughout the infection cycle, suggesting that replication of FMDV was inhibited at RNA synthesis.

### Persistent infection was re-established when infected these isolated FMDV-negative cells with FMDV-Op

**Figure 2:**
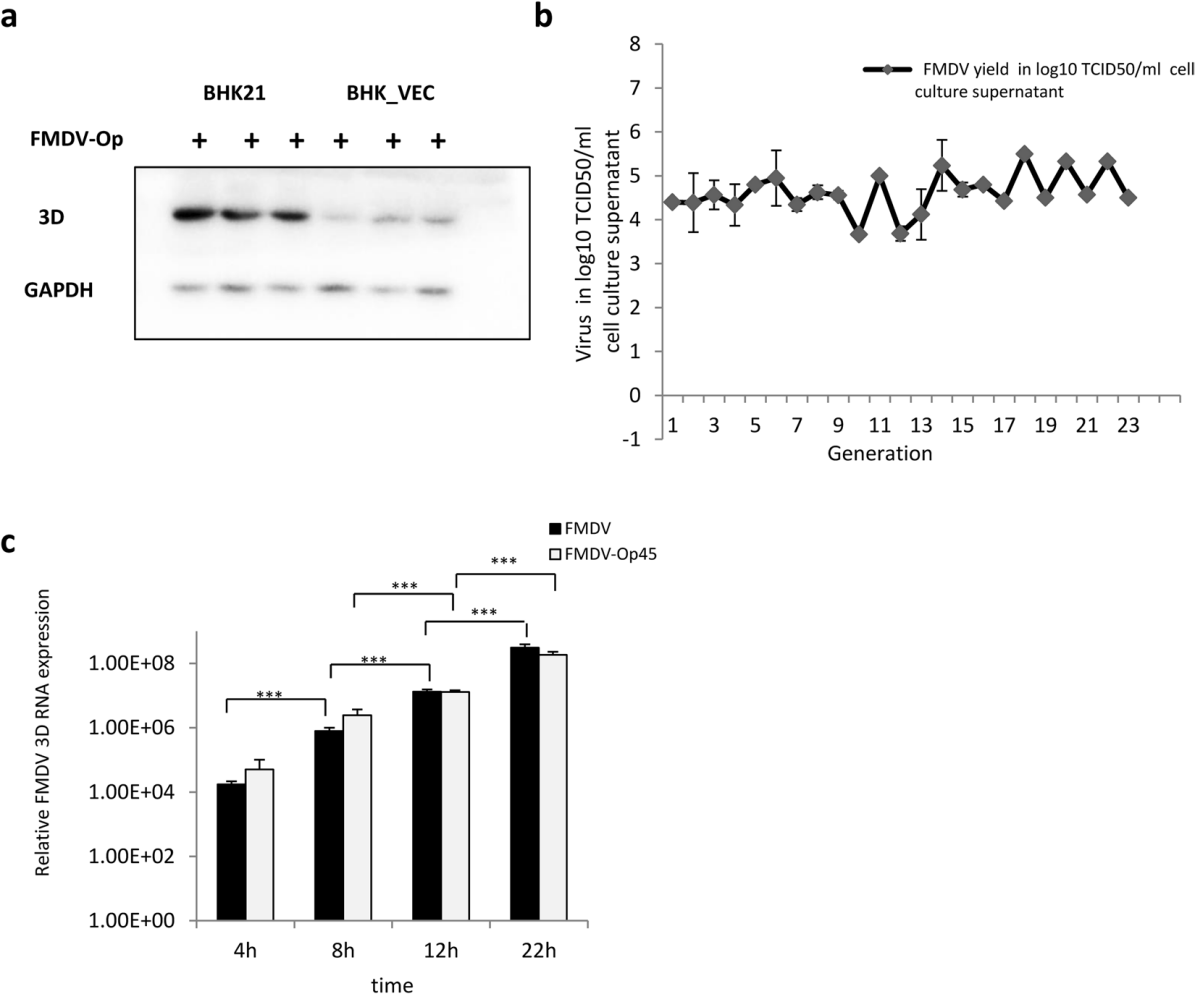
BHK-VECs resisted infection of FMDV-Op. (**a**) BHK-21 cells and BHK-VECs were infected with FMDV-Op at 2 × 10^−4^ PFU/cell for 24 h. Whole-cell extracts were analyzed by western blotting using rabbit polyclonal anti-3D-specific or mouse monoclonal anti-GAPDH antibodies. (**b**) BHK-VECs were infected with FMDV-Op at 2 × 10^−4^ PFU/cell. Infected cells were subcultured, and the secreted infectious virus in the supernatant of each generation was measured by titration assay. (**c**) BHK-21 cells were infected with FMDV or FMDV-Op at 2 × 10^−4^ PFU/cell. Intracellular RNA was isolated for quantification of intracellular virus RNA by qRT-PCR at the indicated time points. ***p < 0.001.

During persistent FMDV infection, host cells blocking viral RNA synthesis evolve to resist FMDV infection. However, their role in persistent infection is unclear. Because FMDV infection is usually accompanied by lysis of host cells, this raises the possibility that FMDV-negative cells are better able to survive in the virus infection process, which may be conducive to the establishment of persistent FMDV infection. Because FMDV could not be amplified effectively and was gradually cleared with the passage of infected BHK-VECs, we inoculated BHK-VECs with FMDV-Op to confirm our hypothesis. We found that infection of BHK-VECs with FMDV-Op did not result in significant cytopathology (data not shown) and allowed only weak viral RNA replication in infected BHK-VECs, in contrast to infected BHK-21 cells (Fig. 2a). By subculturing infected BHK-VECs and measuring the secreted infectious virus in the supernatant of each generation by titration assay, we found that persistent infection was re-established when BHK-VECs were infected with FMDV-Op, as these cultures produced the expected virus levels and could be passaged over 20 generations (Fig. 2b). However, we could not subculture infected BHK-21 cells (data not shown).

These results suggest that FMDV-Op also evolved to overcome the blockade of intracellular viral replication in BHK-VECs. To clarify its role in persistent infection, BHK-21 cells were inoculated with FMDV or FMDV-Op at 2 × 10^−4^ PFU/cell, and intracellular RNA was isolated at different time points post-infection to assess the kinetics of replication of virus RNA using qRT-PCR. We found that infection of BHK-21 cells with FMDV-Op resulted in a significant cytopathic effect (data not shown), consistent with that of FMDV infection, providing evidence of a lytic infection. In parallel experiments, the kinetics of virus RNA replication in BHK-21 cells exhibited no significant changes (Fig. 2c). These results suggest that the evolved viral mutants have the ability to replicate normally in host cells and do not play a decisive role in the establishment of persistent FMDV infection. As our persistently infected cell lines were derived from a single infected cell^31^, these cell-divided FMDV-free cells suggest a mechanism by which persistent FMDV infection is established and maintained. That is, the establishment of persistent FMDV infection is not due to the generation of defective viral particles or other replication-defective mutations during viral infection. Instead, it is due to host cells rapidly dividing into large numbers of evolved cells with increased resistance to the virus, which limits proliferation of the virus and in turn makes the replication of virus and proliferation of host cells more likely to achieve dynamic equilibrium. This facilitates the establishment of persistent infection.

### Global changes of the gene expression profile in the isolated FMDV-negative cells

The transcriptome is the complete set of transcripts in cells and their corresponding quantity at specific developmental stages or under specific physiological conditions. Deciphering the transcriptome is necessary for comprehending functional components of the genome, revealing the molecular composition of cells and tissues, and understanding cellular functions^32^. To understand the genetic basis of BHK-VEC resistance to FMDV infection, we compared global changes in gene expression profiles between BHK-VECs and BHK-21 cells using RNA-seq. For each culture, three independent biological replications were performed, and data were submitted to GEO (accession number GSE93045). In total, more than 41 million 100-bp paired-end reads (41,606,510 to 60,698,628 paired-end reads per sample) were obtained, of which nearly 75% from BHK-21 cells and 77% from BHK-VECs were mapped to the reference genome. The base quality of RNA-seq reads was checked and analyzed using the percentage of Q30% and guanine-cytosine content; we found that our data were of high quality, and each sample had a relatively high sequencing coverage range that was sufficient for downstream analysis (Supplementary Table S2).

**Figure 3:**
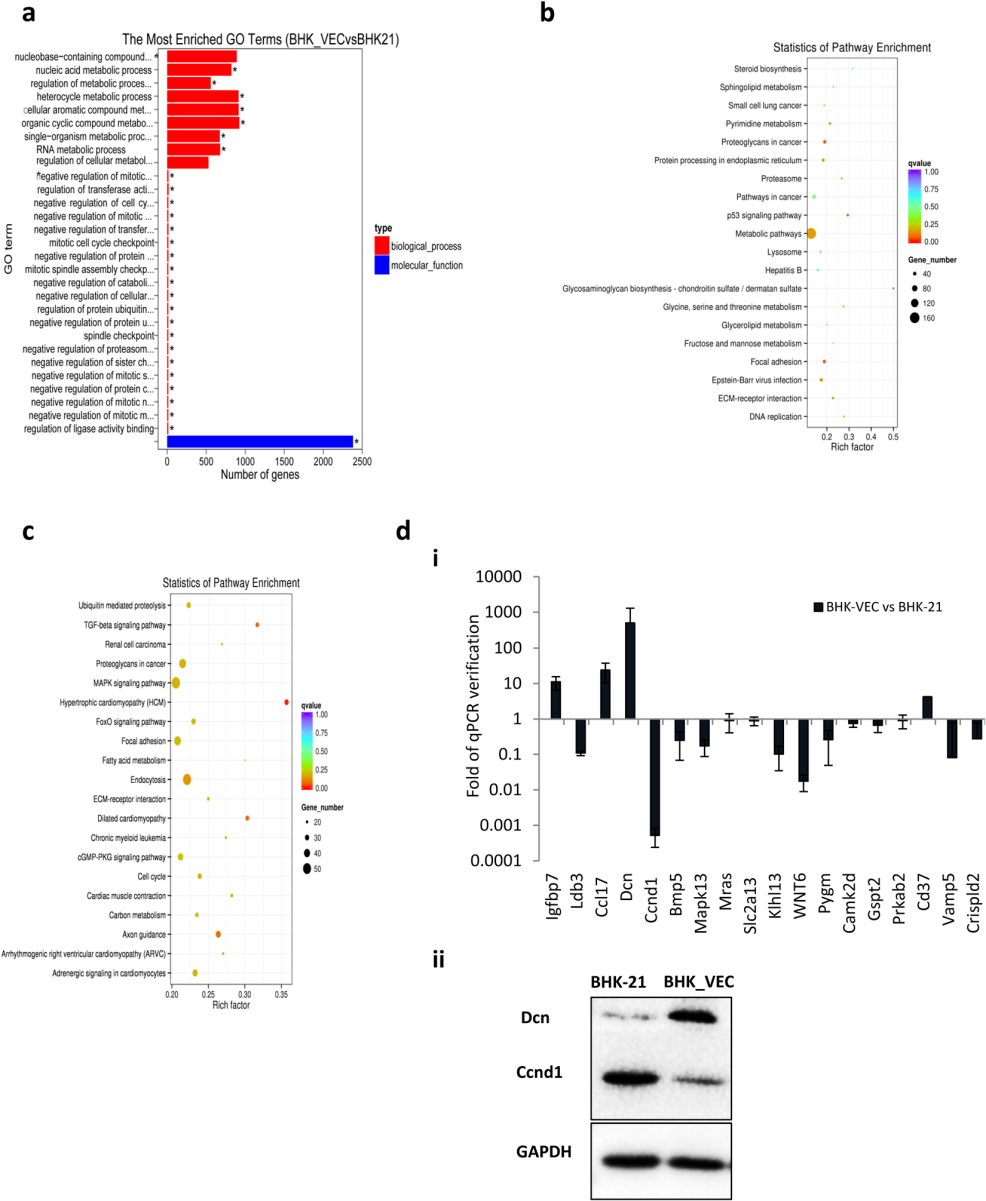
Analysis of DEGs in BHK-VECs. (**a**) GO enrichment analysis of DEGs in BHK-VECs. We included the top 30 most significant GO categories of DEGs (*p < 0.05). KEGG pathway enrichment analysis was performed for differentially expressed (**b**) up-regulated and (**c**) down-regulated genes in BHK-VECs. The graph depicts the most significant 20 enriched pathway entries. (**d**) Validation of DEGs in BHK-VECs by (i) qRT-PCR or (ii) western blotting. For all qRT-PCR and western blot validations, GAPDH served as the internal reference gene to normalize data.

Comparing resistant samples (BHK-VECs) with susceptible samples (BHK-21 cells), a total of 4,686 differentially expressed genes (DEGs) were identified, including 2,168 up-regulated and 2,518 down-regulated genes (Fig. S2a). Gene ontology (GO) functional enrichment analysis indicated that these genes were primarily enriched in categories related to metabolic processes (e.g., nucleic acid metabolic, RNA metabolic, nucleobase-containing compound metabolic, heterocycle metabolic, cellular aromatic compound metabolic, organic cyclic compound metabolic processes), cell division and cell cycle (e.g., negative regulation of mitotic cell cycle and cell cycle phase transition, mitotic cell cycle checkpoint, mitotic spindle assembly checkpoint, negative regulation of sister chromatid segregation, negative regulation of mitotic nuclear division, mitotic metaphase/anaphase transition), cellular protein catabolic processes (e.g., negative regulation of protein modification process and protein ubiquitination, negative regulation of proteasomal ubiquitin-dependent protein catabolic process, negative regulation of transferase activity, regulation of ubiquitin-protein transferase activity, regulation of ubiquitin-protein ligase activity involved in mitotic cell cycle), and binding processes (Fig. 3a; Supplementary Dataset 1). For up-regulated genes, there were no significantly enriched GO terms (entries with *q* < 0.05 were considered significant enrichment terms). For down-regulated genes, the top 20 enriched GO terms were involved mostly in metabolic processes (e.g., nucleic acid and RNA metabolic) and transcription regulation (e.g., regulation of nucleic acid-templated transcription and regulation of transcription, DNA-templated; Fig. S2b). As modifications of host cell metabolic processes by the virus, particularly nucleic acid metabolism, are essential for viral RNA synthesis, viral transcription, and alteration of host cell RNA processing^33^, this raises the possibility that blocks in these processes occur during FMDV infection in BHK-VECs.

To further explore the antiviral mechanism of BHK-VECs, we conducted KEGG pathway enrichment analysis. We found significant enrichment of DEGs in BHK-VECs as compared with BHK-21 cells in three KEGG pathways (corrected *q* < 0.05; Fig. S2d): proteoglycans in cancer, focal adhesion, and extracellular matrix-receptor interaction. Up-regulated DEGs were mainly involved in metabolic processes (e.g., glycosaminoglycan biosynthesis-chondroitin sulfate/dermatan sulfate, pyrimidine metabolism, glycine, serine and threonine metabolism, sphingolipid metabolism, steroid biosynthesis, glycerolipid metabolism, fructose and mannose metabolism and selenocompound metabolism), viral infection (e.g., Epstein-Barr virus infection, hepatitis B), protein catabolic process (e.g., protein processing in endoplasmic reticulum, proteasome and lysosome), and signal transduction (e.g., p53 and PI3K-Akt signaling pathways; Fig. 3b). However, most down-regulated DEGs were involved in signal transduction (e.g., TGF-beta, MAPK, FoxO, cGMP-PKG, Ras, and calcium signaling pathways), endocytosis, and cell cycle (Fig. 3c), suggesting that changes in these pathways may contribute to the resistance of BHK-VECs to FMDV infection.

To confirm the authenticity of our RNA-seq results, we selected several host genes that were differentially expressed in BHK-VECs for qRT-PCR or western blotting (Fig. 3d). We found that qRT-PCR data were highly positively correlated with RNA-seq data (*r* = 0.9779; Fig. S2c; see primer information in Supplementary Table S3). Furthermore, the protein expression of Dcn was up-regulated whereas that of Ccnd1 was down-regulated in BHK-VECs, consistent with RNA-seq DEG analysis. This suggests that our RNA-seq data are credible and can be used as a general positive reference for expression profiling studies.

### Alternative splicing induced in these isolated FMDV-negative cells

**Figure 4:**
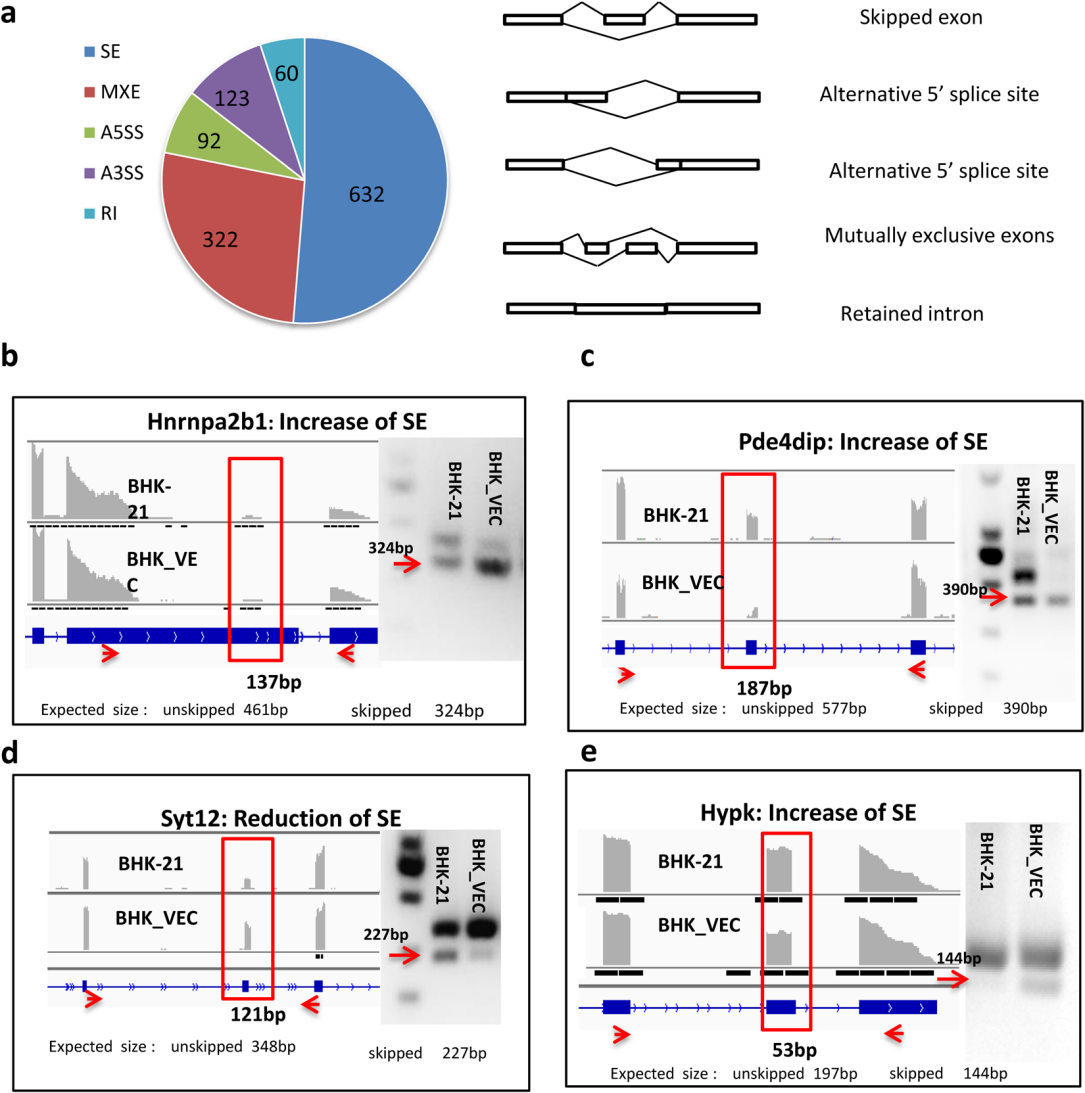
AS induced in BHK-VECs. (**a**) Summary of significant differential AS events in BHK-VECs compared with BHK-21 cells. The intron-exon structure involved in each splicing pattern is shown. (**b**–**e**) Verification of AS events by RT-PCR. Visualization of RNA-seq reads was first performed by IGV. Then, RT-PCR was performed using specific primers located upstream and downstream of SE events. The y-axis shows the number of mapped reads, and the red arrows at the bottom show the target area for primer amplification. Hnrnpa2b1, Pde4dip, and Hypk showed increased SEs in BHK-VECs as shown by a larger number of products of 324, 390, and 144 bp. Syt12 showed a decrease in SEs in BHK-VECs as shown by a larger number of products of 227 bp.

AS of mRNA precursors is a powerful and universal mechanism that regulates gene expression and increases diversity of the transcriptome^34,35^. AS plays a decisive role in generating protein diversity, regulating development and growth, and controlling responses to stimuli^36,37^. To determine whether AS is induced in BHK-VECs and plays a role in their resistance to FMDV infection, we investigated differences in splicing patterns between resistant samples (BHK-VECs) and susceptible samples (BHK-21 cells). We found 1,229 significant AS events in 932 genes in BHK-VECs compared with BHK-21 cells, including 632 skipped exons (SE), 92 alternative 5′ splice sites (A5SS), 123 alternative 3′ splice sites (A3SS), 322 mutually exclusive exons (MXE), and 60 retained introns (RI). SE was the major splicing pattern that occupied half of the total AS event, and the second largest group was the MEX, which suggests that the resistant samples are more inclined to take the SE pattern (Fig. 4a). Notably, most genes (n = 748) occurred only once in the AS event; however, there were 124 genes that occurred twice, 34 that occurred three times, 15 that occurred four times, 6 that occurred five times, 4 (KIAA1191, CD44, Nfya, and Cnbd2) that occurred six times, 1 (Acsl3) that occurred seven times, and 1 (Map4k4) that occurred 10 times in AS events.

To confirm the AS events, we validated four SE splicing patterns in four genes—heterogeneous nuclear ribonucleoprotein A2/B1 (Hnrnpa2b1), phosphodiesterase 4D interacting protein (Pde4dip), synaptotagmin like 2 (Sytl2), and huntingtin interacting protein K (Hypk)—using primers located upstream and downstream of the SE event (Supplementary Table S4). We found that Hnrnpa2b1, Pde4dip, and Hypk exhibited increased splicing in BHK-VECs, as RT-PCR detected increased ratios of splice products with fragment sizes of 324, 390, and 144 bp, respectively, confirming the increase in SEs (arrows in Fig. 4b–e). However, Sytl2 showed reduced splicing in BHK-VECs, with a decreased ratio of splice products with a fragment size of 227 bp and an increased ratio of unspliced products with a fragment size of 348 bp (Fig. 4d). These RT-PCR results are consistent with our RNA-seq results, thus validating the analysis.

To understand the functions involved in these AS events, we performed GO enrichment analysis. The results indicate that AS changes were concentrated in cellular component (2 terms, 324 genes), cellular metabolic processes (12 terms, 534 genes), transcriptional regulation (1 terms, 87 genes), protein modification process (2 terms, 194 genes), cell protein localization (1 terms, 139 genes), and chromatin modification (2 terms, 77 genes; *p* < 0.05; Fig. S3a), suggesting that AS plays an important role in regulating these processes and may quickly produce variations in different functions in response to persistent FMDV infection. Only two KEGG pathways were significantly enriched: amino sugar and nucleotide sugar metabolism and the lysine degradation pathway, and numerous genes were enriched in metabolic pathway categories (Fig. S3b), indicating that host cells were more inclined to exhibit AS events in metabolic processes in response to persistent FMDV infection.

When comparing the AS genes with DEG genes, we found that 288 of the AS genes were also differentially expressed, comprising only 7% of the DEGs (Fig. S3c). This suggests that the differential expression of genes in BHK-VECs does not depend on the occurrence of AS; rather, AS and differential gene expression complement each other as two separate regulatory mechanisms in BHK-VECs.

### Changes in gene expression patterns of innate and adaptive immunity-related genes in isolated FMDV-negative cells

**Figure 5:**
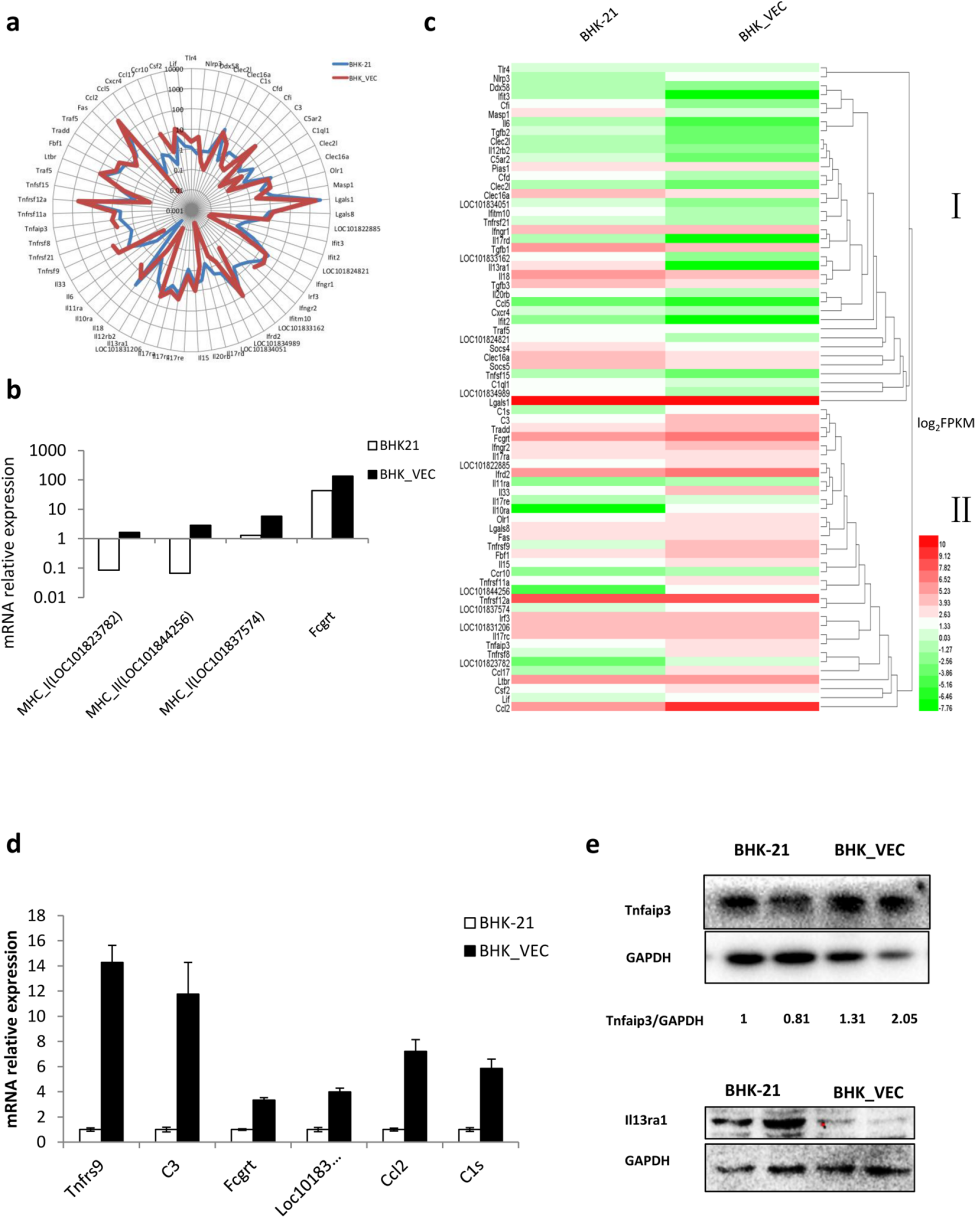
Differential expression of immunity-related genes in BHK-VECs. (**a**) Differential expression of innate immunity-related genes between BHK-21 cells and BHK-VECs were artificially chosen for expression analysis. The x-axis is the periphery of the circle showing the names of genes, and the y-axis is the radius of the circle showing the corresponding FPKM values. The farther away from the center of the circle, the higher the expression of the gene. (**b**) Comparison of adaptive immunity-related gene expression between BHK-21 cells and BHK-VECs. (**c**) Hierarchical clustering heatmap of differentially expressed immunity-related genes between BHK-VECs and BHK-21 cells. The color range from green to red corresponds to a low to high abundance of gene expression, respectively. (**d**,**e**) Validation of differentially expressed immunity-related genes in BHK-VECs by qRT-PCR or western blotting. GAPDH served as the internal reference gene to normalize data.

An effective immune response is essential for the timely removal of virus from infected cells. Earlier studies in persistently FMDV-infected cultures show that many immunity-related genes are differentially expressed in the carrier state and that host cells show impaired cell-mediated immunity^28,38^. To characterize the immune response of BHK-VECs and determine changes in host cell immune response to persistent FMDV infection, we observed the expression patterns of innate and adaptive immunity-related genes. Based on previous reports^39,40^, genes related to pattern recognition receptors, antimicrobial peptides, complement molecules, lectin family members, cytokines in innate immunity, interleukin-1 family members and receptors, tumor necrosis factor, chemokines, and the major histocompatibility complex were chosen for gene expression analysis. We found that 73 immunity-related genes were differentially expressed: 69 innate immunity-related genes and 4 adaptive immunity-related genes. Moreover, the average expression level of these genes in BHK-VECs was higher than that in BHK-21 cells (Fig. 5a,b), indicating that when challenged with FMDV, BHK-VECs may mount a stronger defensive than BHK-21 cells. When differentially expressed immunity-related genes between libraries were clustered, we note that genes in cluster II were more highly expressed in BHK-VECs (Fig. 5c), suggesting that they are vital to the antiviral response.

To confirm the differential expression of these immunity-related genes between BHK-VECs and BHK-21 cells, we performed qRT-PCR or western blotting. The qRT-PCR results show that the expressions of Tnfrs9, C3, Fcgrt, Loc101837574, Ccl2, and C1s were up-regulated in BHK-VECs. Furthermore, the protein expression of Tnfaip3 was up-regulated whereas that of Il13ra1 was down-regulated in BHK-VECs, consistent with results of RNA-seq (Fig. 5d,e).

### Down-regulation of MAPK signaling pathways in isolated FMDV-negative cells

**Figure 6:**
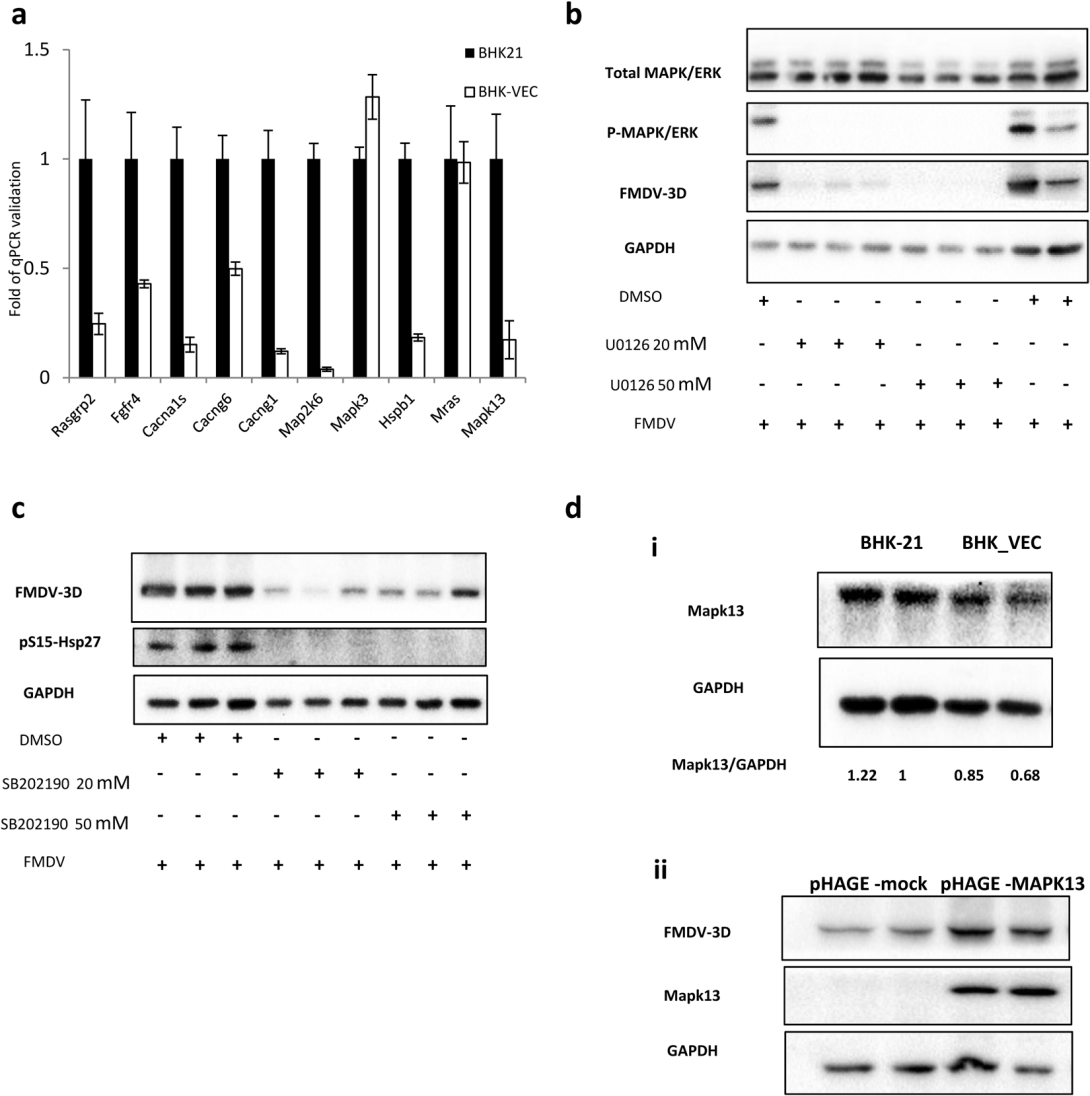
Differential expression of MAPK signaling-related genes in BHK-VECs. (**a**) qRT-PCR validation of DEGs related to MAPK pathways in BHK-VECs. GAPDH served as the internal reference gene to normalize data, and three biologically independent replicates were performed. (**b**,**c**) Effect of MAPK/ERK or p38/MAPK inhibition on replication of FMDV in BHK-21 cells. BHK-21 cells were pre-incubated (1 h) with DMSO, (**b**) 20 or 50 mM U0126, or (**c**) 20 or 50 mM SB202190 and then infected with FMDV at 2.5 × 10^−4^ PFU/cell for 24 h in the presence of DMSO, U0126, or SB202190. Protein extracts were examined using western blotting with FMDV 3D-specific or the indicated antibodies; the effectiveness of inhibition was monitored by detecting the phosphorylation of inhibitor-specific target protein (P-MAPK/ERK and pS15-Hsp27). (**d**) (i) p38δ (MAPK13) was down-regulated in BHK-VECs compared with BHK-21 cells, and (ii) overexpression of MAPK13 genes in BHK-21 cells promoted the replication of FMDV.

Mitogen-activated protein kinase (MAPK) is an important signal transporter that transforms many extracellular signals into gene expression changes and plays an important role in regulating cell proliferation, differentiation, apoptosis, and immune response^41,42^. Previous studies shows that MAPK pathways are activated by many viral infections and are essential for virus multiplication^43–47^. Based on our KEGG pathway analysis of DEGs between BHK-VECs and BHK-21 cells, we found that the MAPK signaling pathway was a dominant down-regulated pathway (Fig. 3c), and several genes that are involved in the ERK/MAPK and p38 MAPK pathways, such as MKK6, p38 delta, ERK, and Ras, were significantly down-regulated in BHK-VECs (Fig. S4). To confirm this finding, we analyzed the differential expression of 10 genes associated with MAPK signaling pathways using qRT-PCR. We found that most of these MAPK signaling pathway-related genes were down-regulated in BHK-VECs, except for MAPK3 and Mras (Fig. 6a).

To test whether this down-regulation of MAPK pathways accounts for the resistance of BHK-VECs to FMDV infection, we artificially suppressed MAPKs to determine their effect on viral replication. In triplicate experiments, we infected BHK-21 cells in the presence of different concentrations of U0126 (a highly specific ERK inhibitor), SB202190 (a potent and selective inhibitor of p38/MAPK), or DMSO and then performed FMDV 3D-specific western blotting to detect the amount of virus 24 h post-infection. The effectiveness of inhibition was monitored by detecting the phosphorylation of inhibitor-specific target protein^48,49^. We found that both inhibitors prevented phosphorylation of the corresponding target (P-MAPK/ERK, pS15-Hsp27) and that virus-infected cells treated with U0126 or SB202190 contained markedly lower amounts of virus compared with cells treated with DMSO (Fig. 6b,c). Next, we performed a single-cycle growth curve experiment to examine whether down-regulation of MAPK signaling pathways inhibited virus replication. BHK-21 cells were infected with FMDV in the presence of 20 mΜ U0126, SB202190, or DMSO, and intracellular RNA was isolated at different time points post-infection to quantify intracellular virus RNA using qRT-PCR. We found that intracellular FMDV RNA levels in BHK-21 cells were significantly inhibited by U0126 and SB202190 at 6 h and reached a peak at 22 h after infection (Fig. S5), indicating inhibition of virus replication. These results suggests that both ERK/MAPK and P38/MAPK signaling pathways are necessary for the proliferation of FMDV.

Considering that the mechanism by which BHK-VECs resist FMDV infection is a complex process involving many changes in host cells, we further explored the effects of host cells on FMDV infection by overexpressing MAPK signaling pathway-related genes in BHK-21 cells. We first verified the down-regulation of MAPK13 (p38 delta) in BHK-VECs by western blotting (Fig. 6di). Next, to investigate the effect of MAPK13 on viral infection, we established a stable MAPK13-overexpressing cell line (pHAGE-MAPK13) from BHK-21 cells based on a lentivirus vectors packaging system. The empty vector pHAGE-mock was used as a negative control. We infected cultures of the same numbers of pHAGE-MAPK13 and pHAGE-mock cells with 2.5 × 10^−4^ PFU/cell FMDV and harvested cells and analyzed cell lysates by western blotting for FMDV 3D protein 24 h later. We found that overexpression of the MAPK13 gene in BHK-21 cells promoted the infection of FMDV (Fig. 6dii), suggesting that downregulation of MAPK13 in BHK-VECs contributes to their resistance to FMDV infection.

In summary, we isolated evolved host cells from persistently FMDV-infected cell lines and revealed their important role in the establishment of persistent FMDV infection. Using RNA-seq, we obtained a relatively complete transcriptome dataset for persistently infected host cells while excluding the effect of viral replication on gene expression. Comparing the evolved host cells (BHK-VECs) with parental cells (BHK-21 cells), we found the differential expression of many genes involved in cell metabolism, cell cycle, and protein metabolism and the induction of 1,229 AS events, particularly SE events. Moreover, BHK-VECs exhibited a stronger response of immune defense-related genes and a weaker MAPK signal response than BHK-21 cells. Our study lays a foundation for further revealing the molecular mechanism of persistent FMDV infection, serves as a basis for further studies of the transcriptome of persistently FMDV-infected host cells, and facilitates the discovery of candidate genes resistant to FMDV infection.

## Materials and Methods

### Cells and virus

The FMDV O serotype virus strain (Akesu/58/2002) was obtained from the Lanzhou Veterinary Research Institute of the Chinese Academy of Agricultural Sciences and was cloned by three successive isolations of plaques formed on BHK-21 cells. BHK-21 cells were a clone of cells provided by the China Center for Type Culture Collection. Persistently infected BHK-21 cells were provided by Dr. Huang. The endogenous virus produced by these carrier cultures was named FMDV-Op followed by the cell passage number (e.g., FMDV-Op48 was the virus shed by culture BHK-Op48)^31^. Cells were cultured in minimum essential medium (MEM, GiBCO, USA) supplemented with 10% heat-inactivated fetal bovine serum (FBS, GiBCO, USA) at 37 °C with 5% CO_2_. Virus titration was determined as previously described using BHK-21 cells^50^. Viral infection and adsorption assays were carried out according to previously published procedures^24,51^. Briefly, BHK21 cells and BHK-VECs were infected with FMDV at 2.8 PFU/cell. At different times post-adsorption at 4 °C, the supernatant was removed rapidly and monolayers washed with cold phosphate-buffered saline (PBS) three times. One mL serum-free MEM was added to the monolayers, and cell suspensions were subjected to three cycles of freezing and thawing and centrifuged for 5 min at 12,000 × g and 4 °C. The supernatant was taken to quantify the bound virus titer by TCID_50_ assay.

MEK1/2 inhibitor U0126 and P38 inhibitor SB202190 were purchased from Selleck. FMDV 3D antibody was donated by Dr. Li. GAPDH antibody, MAPK13 antibody, Dcn antibody, Ccnd1 antibody, and secondary horseradish peroxidase (HRP)-labeled goat anti-rabbit and goat anti-mouse antibodies were purchased from Proteintech. Rabbit polyclonal antibodies against ITGB6, ITGAV, ITGB1, total MAPK/ERK, phospho-MAPK/ERK, Tnfaip3, or Il13ra1 were purchased from Hangzhou Huaan Biotechnology. Phospho-HSPB1-S15 (HSP27) polyclonal antibody was purchased from ABclonal. Secondary FITC-labeled goat anti-rabbit and goat anti-mouse antibodies were purchased from Thermo Fischer Scientific.

### Isolation of FMDV-negative cells by serial dilution and characteristic analysis

BHK-Op48 cells were serially diluted in a 96-well plate at a cell number of 1–10 for minimal inoculation. After culturing for 1–2 weeks, wells with the lowest seeding densities and supernatants with FMDV-negative cells were screened and further expanded in culture. These cells were further examined for FMDV 3D protein using an immunofluorescence antibody test and confirmed to be FMDV RNA-negative by qRT-PCR. To characterize BHK-VECs, cells were infected with FMDV or FMDV-Op, and viral replication was determined by western blotting. In brief, an equal number of BHK-VECs and BHK-21 cells were plated in 24-well plates. When cells reached 80% confluence, they were infected with FMDV at 2.5 × 10^−4^ PFU/cell or FMDV-Op at 2 × 10^−4^ PFU/cell. At the indicated times, whole-cell extracts were analyzed by western blotting using rabbit polyclonal anti-3D and mouse monoclonal anti-GAPDH antibodies. To confirm the successful establishment of persistent infection, the supernatant of each generation of FMDV-Op-infected cells was collected and subjected to TCID_50_ assay.

### Indirect immunofluorescence

Cells grown in 12-well plates were washed twice with PBS and fixed with 4% paraformaldehyde for 30 min followed by permeabilization with 0.5% Triton-X 100 for 10 min. After washing once with PBS, cells were blocked with 2% bovine serum albumin for 30 min, and FMDV 3D was stained using an anti-3D specific polyclonal antibody for 1 h. Cells were then stained with goat anti-rabbit secondary antibody labeled with fluorescein isothiocyanate (FITC) for 30 min. Samples were washed once with PBS and observed with an Olympus IX71 inverted fluorescence microscope coupled with cellSens software.

### Western blot analysis

Proteins were extracted from cells with 2 × SDS buffer and then boiled for 5 min. After centrifuging for 10 min at 10,000 × g, supernatant was subjected to a 10% SDS-PAGE gel for electrophoresis. Afterward, dispersed proteins were electroporated onto a polyvinyl dichloride membrane (Bio-Rad) for western blotting. Prior to the detection of target proteins with specific protein antibody, the transferred membranes were blocked with 5% skim milk for 1 h at room temperature to reduce non-specific hybridization. Excess milk was washed away three times, and immunoblotting was performed with specific protein antibody followed by HRP-conjugated AffiniPure secondary antibody to develop the blotting results. Results were presented using the GelDoc XR System (BIO-RAD), and all experiments were repeated independently three times.

### RNA extraction, library preparation, and Illumina sequencing

BHK-VECs and BHK-21 cells were collected, and RNA was extracted with TRIzol reagent (Takara, China). The integrity and purity of RNA were tested using 1.5% agarose gels, NANO DROP2000 (Thermo), and Agilent 2100. Each sample was subjected to three independent replicates. Subsequent sequencing was performed at Beijing Novogene Illumina Biological Information Technology Co. Ltd. Briefly, mRNA was enriched by Oligo-attached magnetic beads (NEBNextUltraTM RNA library Prep Kit) and then fragmented into 200–400 bp. First-strand cDNA was synthesized using a random hexamer primer and M-MuLV reverse transcriptase with RNase inhibitor. Second-strand cDNA synthesis was subsequently performed using DNA Polymerase I with RNase H. After adenylation of 3′ ends of DNA fragments, NEBNext adaptors with hairpin loop structures were ligated to prepare for sequencing. An AMPure XP system (Beckman Coulter, Beverly, USA) was used to select 200–400 bp cDNA fragments. Then, 3 μl USER Enzyme (NEB, USA) was used with size-selected, adaptor-ligated cDNA at 37 °C for 15 min followed by 5 min at 95 °C before PCR. PCR was performed with Phusion High-Fidelity DNA polymerase, Universal PCR primers, and Index (X) Primer. Lastly, PCR products were purified (AMPure XP System), and library quality was assessed using the Agilent Bioanalyzer 2100 system. After library inspection qualification, HiSeq sequencing was implemented in the different libraries.

### RNA-seq data analysis

After using cutadapt software to remove reads with low quality or those containing adaptor contaminations or Ns, we obtained clean reads. The *Mesocricetus auratus* genome was downloaded from NCBI, and the sequences of clean reads were subjected to genome mapping analysis using TopHat2. Cufflinks was then used for assembly. Both new and known transcripts were identified with Cufflinks 2.1.1, and AS events were analyzed using rMATS software. Visualization of RNA- sequencing reads were performed by Integrative Genomics Viewer (IGV) software. The number of AS events in each sample was estimated separately, and difference analysis was performed. We selected significant AS cases with adjusted *p* ≤ 0.05 for further analysis.

To calculate levels of gene expression, we considered the expected number of fragments per kilobase of the transcript sequence per millions base pair sequencing (FPKM), with FPKM > 1 considered to indicate gene expression. Genes with a fold change of differential expression between samples > 2 and a false discovery rate < 0.05 were considered differentially expressed genes. In addition, cluster analysis was performed to determine the expression patterns of DEGs under different experimental conditions. GO analysis of AS changes and DEGs were carried out using hmmscan and GOseq R packages, respectively. Functions with a corrected *p* < 0.05 were considered enriched GO terms. KEGG enriched analysis was performed to determine which functional pathways contained DEGs using KOBAS v2.0^52,53^.

### Validation of RNA-seq data by qRT-PCR

qRT-PCR was performed to verify RNA-seq data. Primers (Supplementary Table S3) were designed with Primer Premier 6.0 software (Primer, Canada). RNA isolation and cDNA synthesis were processed as previously described. Each qRT-PCR reaction involved 0.25 μL SYBR green dye (Invitrogen), 12.5 μL Premix Taq (Promega), 0.5 μL of each primer (25 μmol/μL), and 6.25 μL H_2_O to a final volume of 25 μL. The amplification reaction was achieved through one cycle at 95 °C for 10 min followed by 40 cycles at 95 °C for 15 s and a final cycle at 60 °C for 60 s in the CFX96TM RT-PCR detection system (BIO-RAD). GAPDH was chosen as an endogenous control to normalize the expression levels of genes, and results were analyzed with the 2^−ΔΔCt^ method. All assays were performed with three independent biological replicates. An FMDV 3D specific primer was used for the quantification of FMDV RNA (Table S3).

### Validation of AS events by RT-PCR

RT-PCR was performed to verify AS events. Primers (Supplementary Table S4) were designed with Primer Premier 6.0 software (Primer, Canada). RNA isolation and cDNA synthesis were processed according to standard protocols. Briefly, 1 μg RNA was reverse transcribed using a random hexamer primer and M-MuLV reverse transcriptase with RNase inhibitor. The RT-PCR reaction involved 12.5 μL Premix Taq (Promega), 0.5 μL of each primer (25 μmol/μL), and 6.5 μL H_2_O to a final volume of 25 μL. The amplification reaction was achieved through one cycle at 95 °C for 10 min followed by 40 cycles at 95 °C for 15 s and a final cycle at 60 °C for 60 s. The products were analyzed using gel electrophoresis.

### Data Availability

The sequencing data generated during the course of this study are available at the Gene Expression Omnibus (GEO) under accession GSE93045. The data that support the findings of this study are available from the corresponding author upon reques.

## Electronic supplementary material

Supplementary information

Supplementary Dataset 1
