# Supplementary material for: Cellular response to persistent foot-and-mouth disease virus infection is linked to specific types of alterations in the host cell transcriptome
Source: Sci Rep. 2018 Mar 22;8:5074. doi: 10.1038/s41598-018-23478-0 (PMC5864922; doi:10.1038/s41598-018-23478-0)
Supplement: Supplementary file 1 — Supplementary information [file 41598_2018_23478_MOESM1_ESM.pdf]

Cellular response to persistent foot-and-mouth disease virus infection is linked to specific types of alterations in the host cell transcriptome

Lingling Han[1], Xiu Xin[1], Hailong Wang[1], Jiadai Li[1], Yao Hao[1], Mingzhen Wang[1], Congyi Zheng[1,2], Chao Shen[1,2*]

[1]State Key Laboratory of Virology, College of Life Sciences, Wuhan University, Wuhan, China

[2]China Center for Type Culture Collection, Wuhan University, Wuhan, China

*Correspondence should be addressed to:

Dr. Chao Shen

E-mail: shenchao@whu.edu.cn

Tel: 86-27-68752093

Fax: 86-27-68754833

List of Supplementary Materials

Table S1. The FMDV or FMDV-Op in BHK_VEC, BHK21 and BHK_OP54 were detected by qRT-PCR.

Table S2. summary statistics of RNA-Sequencing data.

Table S3. Primers and probes for quantitative RT-PCR.

Table S4. Sequences of primers used for RT-PCR.

Figure S1. Indirect immunofluorescence for detection of FMDV or FMDV-Op in BHK_Op and BHK_VEC. FMDV 3D-specific antibodies stained negative control BHK-21 (b), BHK_Op43 (a), BHK_VEC (d), and positive control BHK-21 cells that were infected with FMDV at 0.06 PFU/cell for 12 hours. The 3D positive is in green (Alexa 555), and the lower row is the cell morphology pictures of each cell line (×100).

Figure S2. Analyses of differentially expressed genes in BHK_VEC. (a) Differentially expressed genes were determined in RNA levels in BHK_VEC compared with BHK-21(FPKM value ≥ 1 and p_value ≤ 0.05). A total of 4686 genes were differentially expressed including 2168 upregulated genes and 2518 down-regulated genes. (b) GO enrichment analysis of differentially down-regulated expressed genes in BHK_VEC; based on p_Value, we included the top 30 most significant GO categories in the DEGs (*$p < 0.05$). (c) Correlation analysis between qRT-PCR and RNA-seq data. (d) KEGG pathway enrichmen analysis of DEGs in BHK-VECs. We included the top 30 most significant KEGG pathway categories of DEGs .

Figure S3. GO and KEGG pathway analyses of AS changes in BHK-VEC. (a) GO enrichment analysis of genes with differentially alternative splicing in BHK_VEC compared with BHK-21. The most significant 20 GO terms of enrichment are shown. (b) KEGG pathway enrichment analysis of genes involved in differentially alternative splicing in BHK_VEC vs BHK-21. The most significant 10 GO terms of enrichment are shown. (c) Overlap of the genes involved in differentially expressed and differentially alternative splicing in BHK_VEC.

Figure S4. Significantly differentially expressed genes identified by KEGG as involved in MAPK signaling[1]. Red: significantly increased expression (fold-change >2); Blue: significantly decreased expression (fold-change <0.5); Green: genes detected in downregulated groups; Gray: unchanged expression.

Figure S5. Effect of MAPK/ERK or p38/MAPK inhibition on the replication of FMDV in BHK-21. BHK-21 cells were pre-incubated (1 h) with DMSO or 20 mM U0126, or 20 mM SB202190, then exposed to 2.5×10-4 PFU/cell FMDV in the presence of U0126 (a highly specific ERK inhibitor) or SB202190 (a potent and selective inhibitor of p38/MAPK) or DMSO for different times. At the indicated time maintained, the intracellular RNA was isolated and intracellular virus RNA numbers were deteced by QRT-PCR analysis. (***$p < 0.001$, *$p < 0.05$.).

Figure S6. As a complement, the full-length blot / gel diagram for indicated figures was shown.

Figure S7. As a complement, the full-length blot / gel diagram for indicated figures was shown. Among them, we can not always detect the background expression of MAPK13 due its low expression in the host cells (MAPK13 can only be detected when the real-time quantitative PCR cycle number is more than 35 CT values). For example, Figure 6d- ii, we did not succeed in detecting MAPK13 in the pHAGE-mock cell line.

Table S1. The FMDV or FMDV-Op in BHK_VEC, BHK21 and BHK_OP54 were detected by qRT-PCR.

cell	CT 值
BHK21	NA
BHK_VEC	NA
BHK_Op54	15.04

Table S2. summary statistics of RNA-Sequencing data.

Sample name	BHK-21_1	BHK-21_2	BHK-21_3	VEC_1	VEC_2	VEC_3
Raw reads	55613610	56411208	57451398	60698628	41606510	43957326
Clean reads	53734426	54484692	55573216	59577214	40942086	43217992
Q30 (%)	94.94	94.92	94.93	94.3	94.55	94.49
GC content (%)	50.17	49.98	49.85	50.93	51.45	51.74
Total mapped	40381924 (75.15%)	40983864 (75.22%)	41907283 (75.41%)	46159447 (77.48%)	31526450 (77%)	33035916 (76.44%)
Uniquely mapped	39973778 (74.39%)	40546457 (74.42%)	41487974 (74.65%)	45683845 (76.68%)	31210654 (76.23%)	32691697 (75.64%)
Reads map to“+”	19963997 (37.15%)	20254923 (37.18%)	20715860 (37.28%)	22832938 (38.32%)	15600284 (38.1%)	16344676 (37.82%)
Reads map to“-”	20009781 (37.24%)	20291534 (37.24%)	20772114 (37.38%)	22850907 (38.36%)	15610370 (38.13%)	16347021 (37.82%)
Non-splice reads	22246041 (41.4%)	22323855 (40.97%)	22891087 (41.19%)	24130499 (40.5%)	16428437 (40.13%)	16700228 (38.64%)
Splice reads	17727737 (32.99%)	18222602 (33.45%)	18596887 (33.46%)	21553346 (36.18%)	14782217 (36.11%)	15991469 (37%)

Table S3. Primers and probes for quantitative RT-PCR.

Gene name	Gene ID	Forward Primer/Probe Sequence (5’-3’)	Reverse primer(5’-3’)
Igfbp7	101839936	TGCTGGTATCTCCTCTAAGTAAG	CTGATGCTGAAGCCTGTCC
Ldb3	101829490	CTACCATCATCCACGCACAG	ACAGCCAAGTCTTTCACAGG
Ccl17	101828037	GTCCAGGGCAGGTCCATC	ACGGTCACAGGCTAAGGC
Dcn	101835832	TGCCAGTGTCATCTAAGAGTG	ATGTTGTGTCAGGTGGAAGG
Ccnd1	101837554	GTATCTACACTGACAACTCTATCC	GTTCTCATCCGCCTCTGG
Bmp5	101824438	ACAGTAGACAGAACACAGGAG	AAGCCAGAAGCCAGAAGC
Mapk13	101824910	GCTCAGGCTCTTGCTCAC	TGCTGTTTCCACTCATCTACG
Mras	101824687	TGAAGCACACGGAGATTGAC	GGAGTACACGATGAGGAAGC
Slc2a1	101823763	GAGCCCAGCAGCAAGAAAG	TGATGACGCCAGTGTTATAGC
Klhl13	101828313	ATGCTGGAACTGTGTATGGAG	GGTCATTGGTGCCTTCTGG
WNT6	101842149	GTGGAGACGATGTGGACTTC	TGCCTGCCTCATTGTTGTG
Pygm	101835714	CGCACACAGCAGCATTAC	TCATCGCAGGCATTCTCC
Camk2d	101833285	AGCCATAGAAGTTCAAGGAGAC	CCACCAGCAAGATGTAGAGG

Gspt2	101825988	GTAGCAGCGACTCAACTCC	GCAGAGGCGAATCCATCC
Cd37	101824219	ACGAGTCTGAGGAGCCCTTG	AGAGGTCAGCGGTCTGTCTC
Vamp5	101823478	TCAGCAAGACAACCAAGACTC	GACCAGCAGCACAATCAGG
Crispld2	101841720	ACGCCGTGTATCTTGTCTG	TCTTGGTAGCATAGGTTGTTCC
GAPDH	106022412	AAGGCCATCACCATCTTCCA	GCCAGTAGACTCCACAACATAC
Tnfrsf9	101829103	CTGCTGCTGTTCCTGATC	CCTCCTCCTTCTTCTTCCT
C3	101842900	AGAAGACGGTGCTGACAAG	TTGACTGGAACTCCTTACTGG
Fcgrt	101825614	AGGAGATGAACACCATTACC	CCACCAAGAAGCCAAGAA
Loc101837574	101837574	TCGGCTACTACAACCAGAG	TCGTAGGCATACTGACTGT
Ccl2	101839392	AACGCTTCTGTGCCTACT	TGCTGCTGGTGATTCTCT
C1s	101823163	AGAACTACAAGGACTGGATACTG	GGCTGGCGGTATGAATGG
Rasgrp2	101840143	CCTCTTCAACAGCGTCTCAC	CCGACAACAGCCATCAGC
Fgfr4	101828736	CCAGCCAGACCAGACCAG	CTTGTGCCGACCGATTAGC
Cacna1s	101835763	CTGGAGTCACCTGTCTTCTTG	GAGGAAGAGCAGGATGAAGG
Cacng6	101835632	ATGATGATGTGGTCTAACTTCTTC	GCCGAGATTCTGCTGTCC
Cacng1	101838313	ATGAGGCAATCGGTGAAGC	GAGGAAGAGCAGGATGAAGG
Map2k6	101833237	TGGTGGACTCTGTTGCTA	GGCTCTTCTACGACTTGTT
Hspb1	101844946	CTGGTGTCCTCTTCCTTGTC	TGGTGATCTCCGATGATTGTG
Mapk3	101841668	GGAGGTGGAGGTGGTGAAG	CGTGGTCGTAGGCAGAGC
3D		GAACACATTCTTTACACCAGGAT	CATATCTTTGCCAATCAACATCAG
3D-Probe		FAM-ACAACCTACCGCCGAGCCAATTC-TAMRA	

Table S4. Sequences of primers used for RT-PCR.

**Primers**	**Sequences**
Hnrnpa2b1-F	5' TGGAAGGAGCCGATACTG 3'
Hnrnpa2b1-R	5' AACTCATCTGTCTACTCACA 3'
Pde4dip-F	5' TGAGTCGTTCTGCTGTGT 3'
Pde4dip-R	5' CTGTGGGTCTGGAGTTAAG 3'
Syt12-F	5' CCTTCACTGCCTGATAAT 3'
Syt12-R	5' GCTGCTGCTAAGTCCTTAC 3'
Hypk-F	5' AGACGGTCCAGGGAGCAA 3'
Hypk-R	5' CATCAGTTGGTTAAGGCAAT 3'

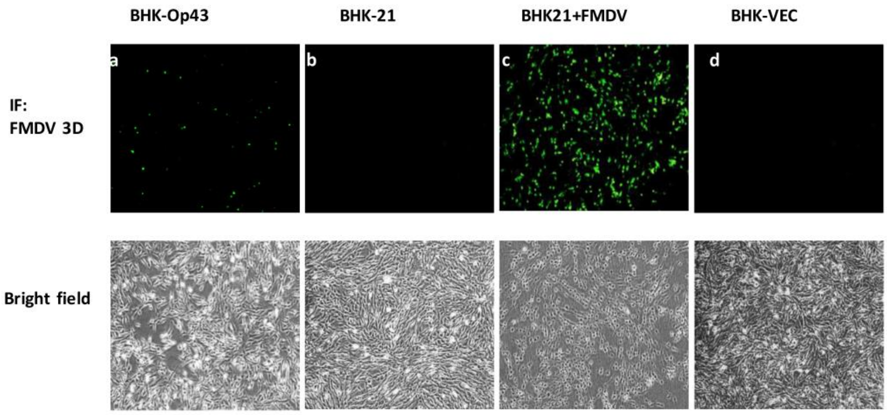

Figure S1. Indirect immunofluorescence for detection of FMDV or FMDV-Op in BHK_Op and BHK_VEC. FMDV 3D-specific antibodies stained negative control BHK-21 (b), BHK_Op43 (a), BHK_VEC (d), and positive control BHK-21 cells that were infected with FMDV at an PFU/cell of 0.06 for 12 hours. The 3D positive is in green (Alexa 555), and the lower row is the cell morphology pictures of each cell line (×100).

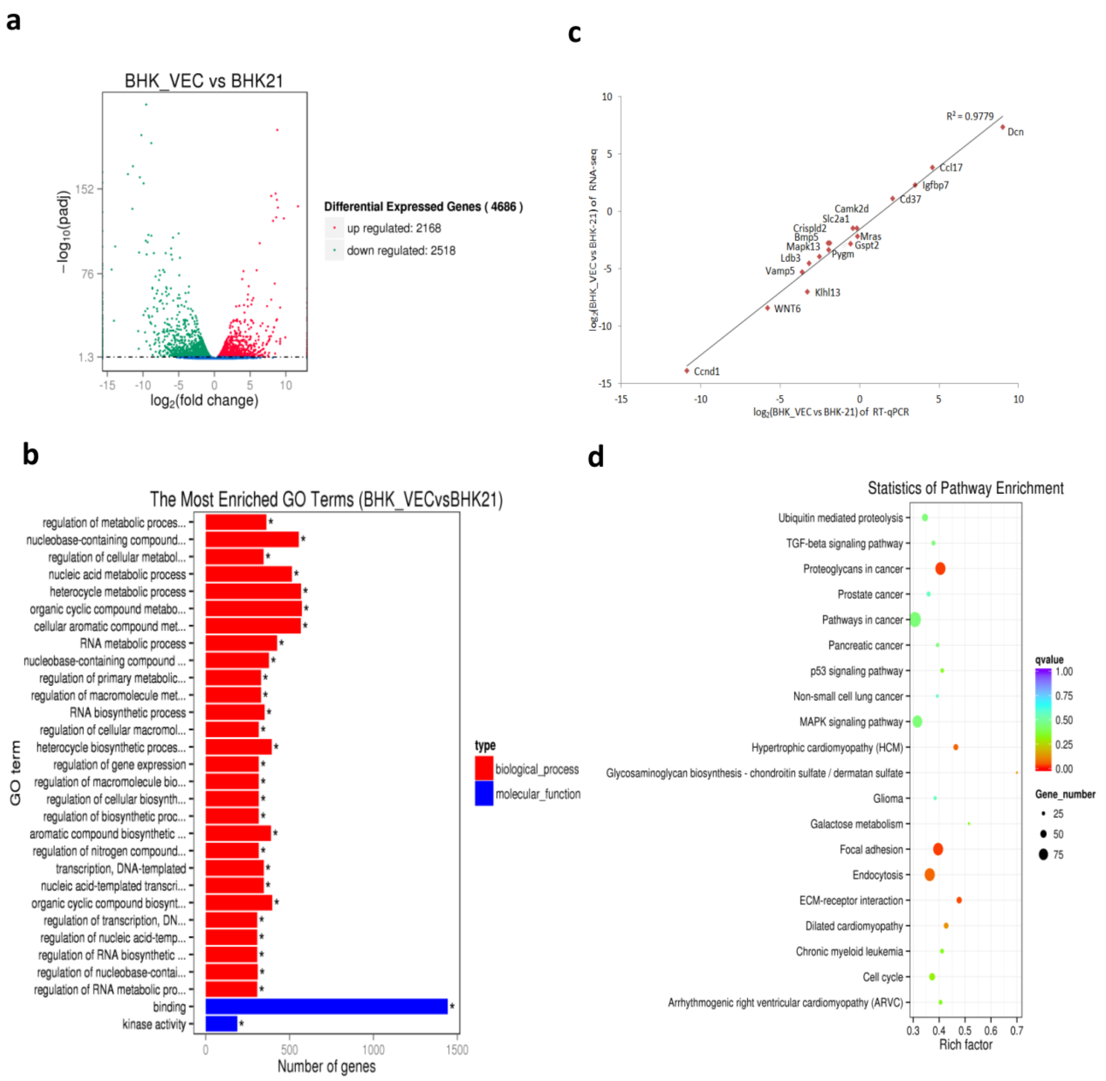

Figure S2. Analyses of differentially expressed genes in BHK_VEC. (a) Differentially expressed genes were determined in RNA levels in BHK_VEC compared with BHK-21(FPKM value ≥ 1 and p_value ≤ 0.05). A total of 4686 genes were differentially expressed including 2168 upregulated genes and 2518 down-regulated genes. (b) GO enrichment analysis of differentially down-regulated expressed genes in BHK_VEC; based on p_Value, we included the top 30 most significant GO categories in the DEGs (*$p < 0.05$). (c) Correlation analysis between qRT-PCR and RNA-seq data. (d) KEGG pathway enrichmen analysis of DEGs in BHK-VECs. We included the top 30 most significant KEGG pathway categories of DEGs .

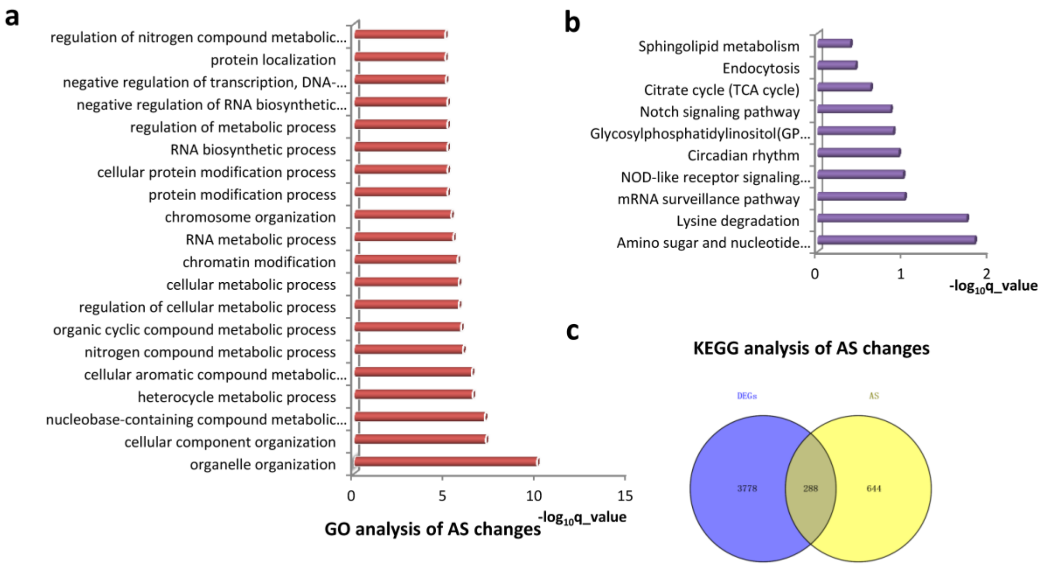

Figure S3. GO and KEGG pathway analyses of AS changes in BHK-VEC. (a) GO enrichment analysis of genes with differentially alternative splicing in BHK_VEC compared with BHK-21. The most significant 20 GO terms of enrichment are shown. (b) KEGG pathway enrichment analysis of genes involved in differentially alternative splicing in BHK_VEC vs BHK-21. The most significant 10 GO terms of enrichment are shown. (c) Overlap of the genes involved in differentially expressed and differentially alternative splicing in BHK_VEC.

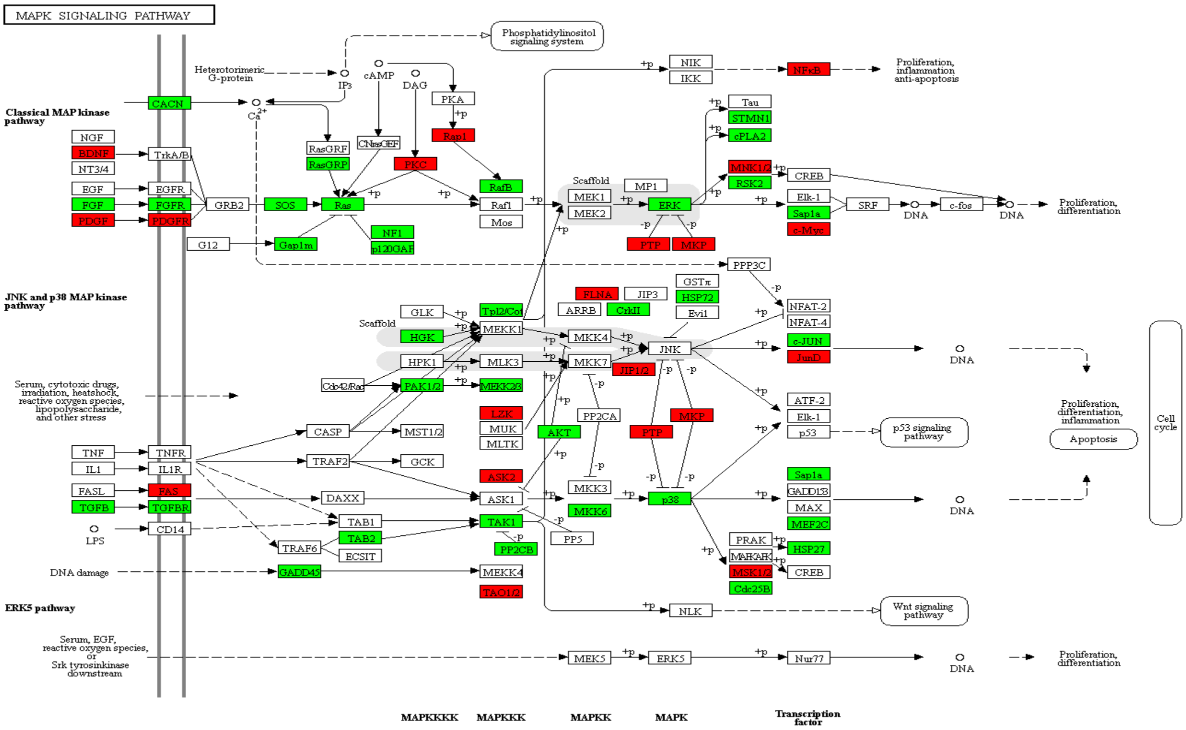

Figure S4. Significantly differentially expressed genes identified by KEGG as involved in MAPK signaling[1]. Red: significantly increased expression (fold-change >2); Blue: significantly decreased expression (fold-change <0.5); Green: genes detected in downregulated groups; Gray: unchanged expression.

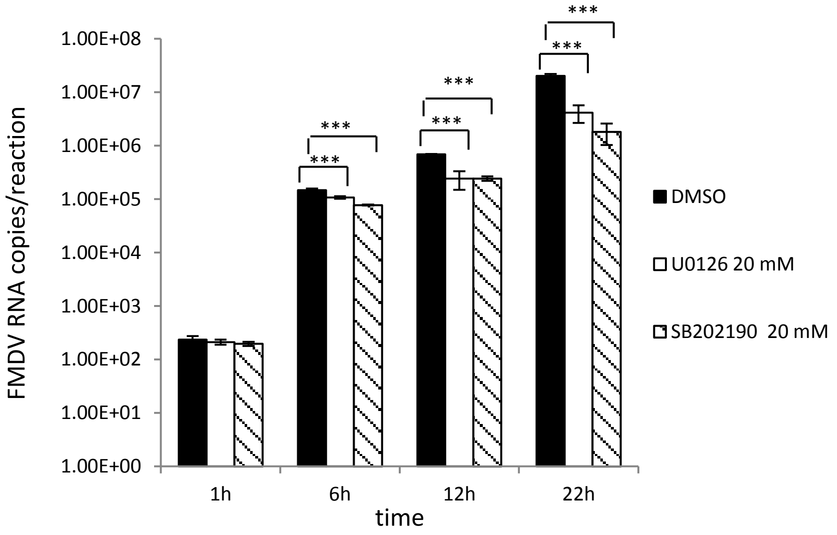

Figure S5. Effect of MAPK/ERK or p38/MAPK inhibition on the replication of FMDV in BHK-21. BHK-21 cells were pre-incubated (1 h) with DMSO or 20 mM U0126, or 20 mM SB202190, then exposed to 2.5×10-4 PFU/cell FMDV in the presence of U0126 (a highly specific ERK inhibitor) or SB202190 (a potent and selective inhibitor of p38/MAPK) or DMSO for different times. At the indicated time maintained, the intracellular RNA was isolated and intracellular virus RNA numbers were deteced by QRT-PCR analysis. (***p < 0.001, *p < 0.05.).

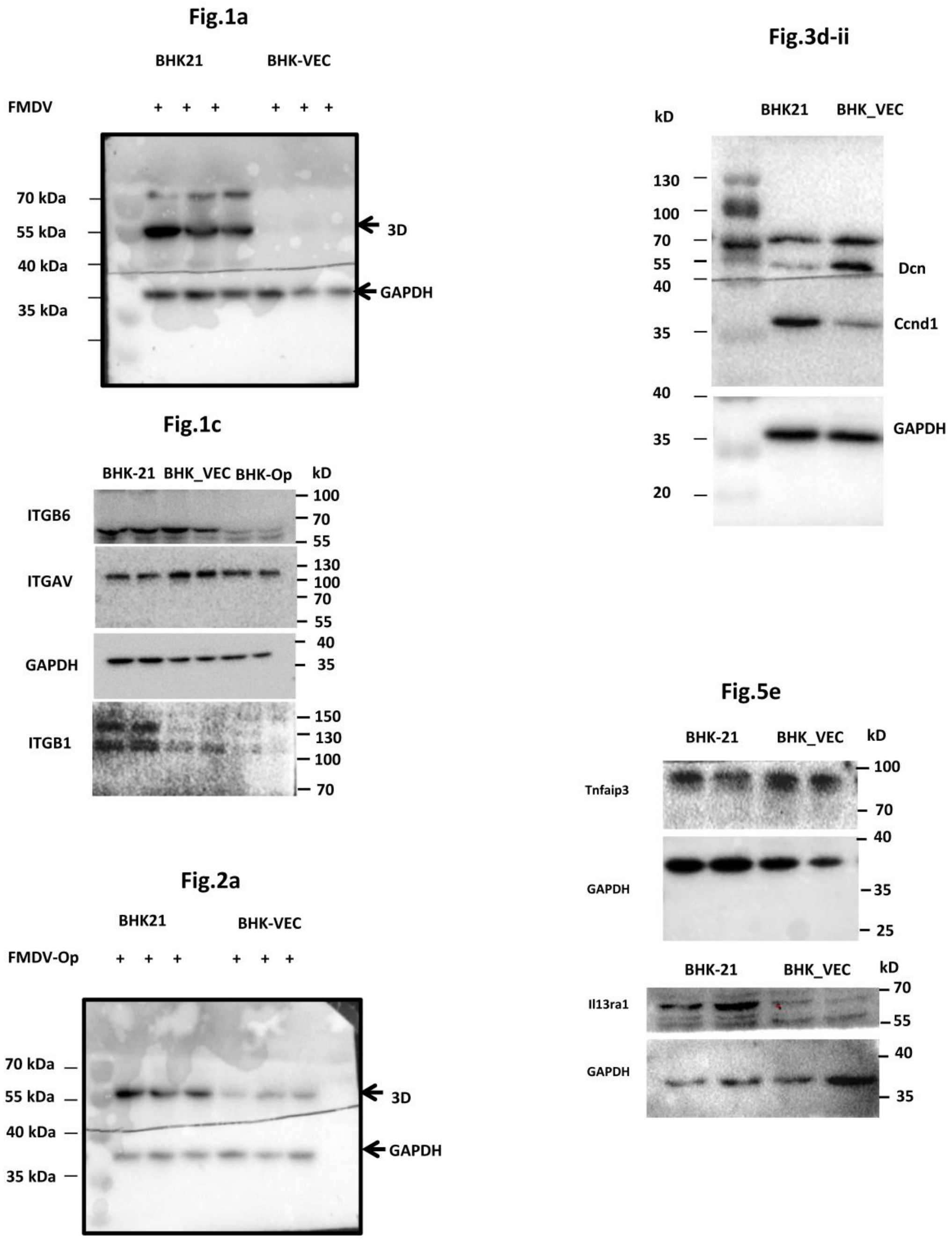

Figure S6. As a complement, the full-length blot / gel diagram for indicated figures was shown.

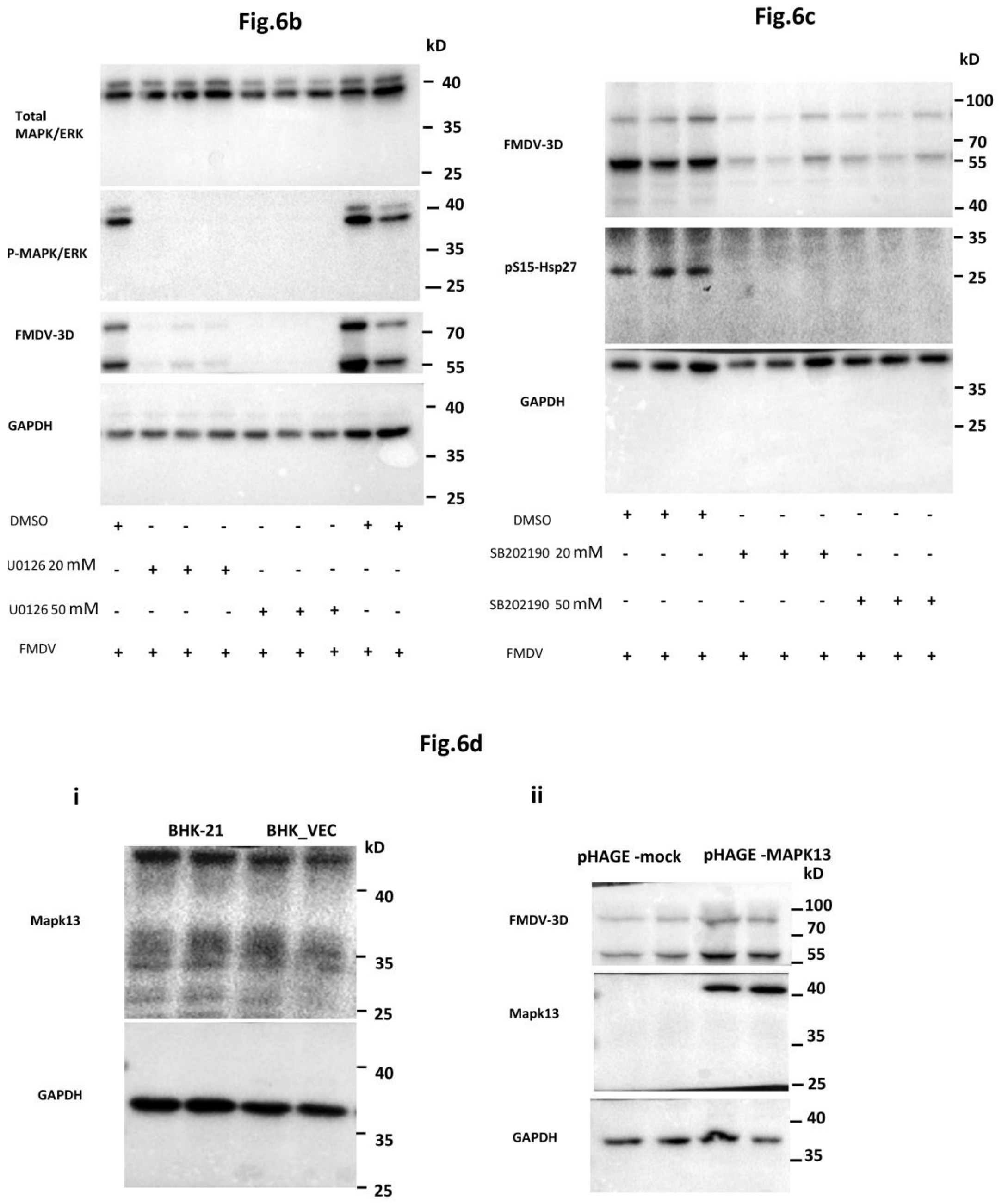

Figure S7. As a complement, the full-length blot / gel diagram for indicated figures was shown. Among them, we can not always detect the background expression of MAPK13 due its low expression in host cells (MAPK13 can only be detected when the real-time quantitative PCR cycle number is more than 35 CT values). For example, Figure 6d-ii, , we did not succeed in detecting MAPK13 in the pHAGE-mock cell line.

References

1 Kanehisa, M., Sato, Y., Kawashima, M., Furumichi, M. & Tanabe, M. KEGG as a reference resource for gene and protein annotation. *Nucleic Acids Res* **44**, D457-462 (2016).
